# Proteomic characterization of paired non-malignant and malignant African-American prostate epithelial cell lines distinguishes them by structural proteins

**DOI:** 10.1186/s12885-017-3462-7

**Published:** 2017-07-11

**Authors:** Jennifer S. Myers, Karin A. Vallega, Jason White, Kaixian Yu, Clayton C. Yates, Qing-Xiang Amy Sang

**Affiliations:** 1 Department of Chemistry and Biochemistry and Institute of Molecular Biophysics, Florida State University, 95 Chieftan Way, Tallahassee, FL 32306-4390 USA; 2 Department of Biology and Center for Cancer Research, Tuskegee University, Tuskegee, AL 36088 USA; 3 Department of Biostatistics - Unit 1411, University of Texas MD Anderson Cancer Center, Houston, TX 77030-1402 USA

**Keywords:** Prostate cancer, RC-77 T/E, African-American cell line model, Comparative proteomics, Differentially expressed proteins, Cancer health disparity, Beta-catenin, Caveolin-1, Integrins

## Abstract

**Background:**

While many factors may contribute to the higher prostate cancer incidence and mortality experienced by African-American men compared to their counterparts, the contribution of tumor biology is underexplored due to inadequate availability of African-American patient-derived cell lines and specimens. Here, we characterize the proteomes of non-malignant RC-77 N/E and malignant RC-77 T/E prostate epithelial cell lines previously established from prostate specimens from the same African-American patient with early stage primary prostate cancer.

**Methods:**

In this comparative proteomic analysis of RC-77 N/E and RC-77 T/E cells, differentially expressed proteins were identified and analyzed for overrepresentation of PANTHER protein classes, Gene Ontology annotations, and pathways. The enrichment of gene sets and pathway significance were assessed using Gene Set Enrichment Analysis and Signaling Pathway Impact Analysis, respectively. The gene and protein expression data of age- and stage-matched prostate cancer specimens from The Cancer Genome Atlas were analyzed.

**Results:**

Structural and cytoskeletal proteins were differentially expressed and statistically overrepresented between RC-77 N/E and RC-77 T/E cells. Beta-catenin, alpha-actinin-1, and filamin-A were upregulated in the tumorigenic RC-77 T/E cells, while integrin beta-1, integrin alpha-6, caveolin-1, laminin subunit gamma-2, and CD44 antigen were downregulated. The increased protein level of beta-catenin and the reduction of caveolin-1 protein level in the tumorigenic RC-77 T/E cells mirrored the upregulation of beta-catenin mRNA and downregulation of caveolin-1 mRNA in African-American prostate cancer specimens compared to non-malignant controls. After subtracting race-specific non-malignant RNA expression, beta-catenin and caveolin-1 mRNA expression levels were higher in African-American prostate cancer specimens than in Caucasian-American specimens. The “ECM-Receptor Interaction” and “Cell Adhesion Molecules”, and the “Tight Junction” and “Adherens Junction” pathways contained proteins are associated with RC-77 N/E and RC-77 T/E cells, respectively.

**Conclusions:**

Our results suggest RC-77 T/E and RC-77 N/E cell lines can be distinguished by differentially expressed structural and cytoskeletal proteins, which appeared in several pathways across multiple analyses. Our results indicate that the expression of beta-catenin and caveolin-1 may be prostate cancer- and race-specific. Although the RC-77 cell model may not be representative of all African-American prostate cancer due to tumor heterogeneity, it is a unique resource for studying prostate cancer initiation and progression.

**Electronic supplementary material:**

The online version of this article (doi:10.1186/s12885-017-3462-7) contains supplementary material, which is available to authorized users.

## Background

Prostate cancer continues to be a substantial burden in the American population. It remains the second leading cause of cancer death among American men, and model-based estimates continue to predict prostate cancer to be most frequently diagnosed among new cancer cases in American men [1]. Prostate cancer is particularly intriguing because of the striking racial health disparity between African-American and Caucasian-American patients. In the most recent data, African-American men have had the highest prostate cancer incidence and mortality of any race and ethnicity in the United States [1]. Race is a significant risk factor for prostate cancer: African-American men are more likely to receive a prostate cancer diagnosis, with a reported incidence rate between 1.5 and 1.86 times higher in African-American men than in Caucasian-American men [1–3]. African-American men are also more likely to receive that diagnosis at a younger age, 3 years younger than Caucasian-American men [4, 5]. Furthermore, prostate cancer mortality is twice as high in African-American men compared to Caucasian-American men [1, 6].

Prostate cancer racial disparities between African-American and Caucasian-American patients often reflect more advanced or aggressive cancer in African-American men. African-American men present with higher grade tumors, report more treatment-related side effects, and have shorter progression-free survival [5]. Men with high-risk prostate cancer were more likely to be African-American, even in patients with low prostate-specific antigen levels [7]. Tumor volumes were reported to be larger in African-American men compared to matched Caucasian-American specimens [8]. Higher Gleason scores and cancer volumes were also reported in African-American men compared to Caucasian-Americans [9]. Gene and microRNA profiling of African-American and Caucasian-American tumor tissue have demonstrated racial variation [10–17]. In light of this, it is increasingly important to study prostate cancer in the context of race, as tumor characteristics have been shown to vary by race. Although socioeconomic factors, treatment choices, comorbidities, and quality of medical care factor into higher incidence and mortality rates, increased prostate cancer-specific mortality is largely attributed to tumor characteristics [18].

One approach to exploring the mechanisms of prostate cancer development and progression is the use of prostate cancer-derived cell lines as in vitro models of the disease. PC-3, DU145, and LNCaP cell lines are popular, well-established, and well-characterized prostate cancer research models [19–21]. The gene and protein expression profiles of these cell lines and their derivatives have also been outlined [19–25]. According to American Type Culture Collection data sheets, PC-3, DU145, and LNCaP cell lines were established from Caucasian prostate cancer patients aged 59 to 69 years old. The PC-3 cell line was established from a prostatic adenocarcinoma metastatic to bone, and PC-3 cells have features common to neoplastic cells and do not respond to androgen [23]. The DU145 cell line was established from a brain metastasis of human prostate carcinoma, and DU145 cells do not express androgen receptors [19, 21]. The LNCaP cell line was established from a supraclavicular lymph node metastatic lesion of prostate adenocarcinoma. While LNCaP cells express androgen receptors and grow in response to androgen, they lose this requirement for growth in later passages [23]. Cell lines derived from non-African-American backgrounds may be less beneficial in providing an understanding of the factors leading to high prostate cancer risk in African-American men. They may also be inadequate for explaining the aggressiveness of prostate cancer in African-American men. However, few prostate cancer models have been established from African-American patients. E006AA is an epithelial cell line with low tumorigenicity derived from cancerous tissue of an African-American patient diagnosed with clinically localized T2aN0M0 prostate cancer [26]. Another cell line, E006AA-hT, which was derived from E006AA cells, is highly tumorigenic [27]. The non-neoplastic RC-165 N cell line was derived from benign tissue of an African-American patient and immortalized by telomerase [28]. MDA PCa 2a and MDA PCA 2b cell lines were derived from a bone metastasis of an androgen-independent cancer from an African-American patient [29]. These cell lines are tumorigenic but have deviated from the androgen insensitive phenotype from which they were derived (i.e., the cells behave differently in vivo and in vitro). None of the above-mentioned models is a malignant and non-malignant pair.

The human malignant and non-malignant immortalized prostate epithelial cell lines RC-77 T/E and RC-77 N/E were established previously from prostate tissue from an African-American patient [30]. This primary tumor was a stage T3c poorly differentiated adenocarcinoma of Gleason score 7. RC-77 cell lines have epithelial character, have functioning androgen receptors, are immortalized, and form a malignant and non-malignant pair. There are few studies on RC-77 cell lines. To date, the RC-77 cell lines have been characterized in terms of miRNA expression, ATP-binding cassette sub-family D member 3 (ABCD3) gene expression, roundabout homolog 1 (ROBO1) mRNA and protein expression, and B lymphoma Mo-MLV insertion region 1 homolog (BMI1) protein levels [17, 31–34]. This work is the only comprehensive proteomic characterization of RC-77 T/E and RC-77 N/E cell lines.

## Methods

### Cell culture and lysis

Both RC-77 N/E and RC-77 T/E cell lines were cultured in Keratinocyte–SFM medium supplemented with bovine pituitary extract and recombinant epidermal growth factor (Life Technologies, Inc., Gaithersburg, MD) in a fully humidified incubator containing 95% air and 5% CO_2_ at 37 °C. After aspirating culture medium, cells were washed twice with phosphate-buffered saline. The washed cells were collected and lysed on ice for 10 min in NP-40 lysis buffer (50 mM Tris-HCl pH 7.2; 150 mM NaCl; 1% Triton X-100; 0.1% sodium dodecyl sulfate; 0.2% sodium deoxycholate in water) containing an EDTA-free protease and phosphatase inhibitor cocktail (Thermo-Pierce, Rockford, IL) at a ratio of 20 μL buffer/500,000 cells. Cell lysates were spun at 14,000 rpm at 4 °C for 10 min. The supernatant was collected and the pellet discarded.

### Mass spectrometry

Cell lysates were desalted on Zeba™ Desalt Spin Columns (Thermo-Pierce, Rockford, IL). Using a ProteoExtract™ All-in-One Trypsin Digestion Kit (Calbiochem, Darmstadt, Germany), vacuum-dried cell lysates were re-suspended, and proteins were extracted into a mass spectrometry-compatible buffer then digested with trypsin. Protein expression was analyzed by high-resolution electrospray tandem mass spectrometry (MS/MS) with an externally calibrated Thermo LTQ Orbitrap Velos mass spectrometer. For each of three biological replicates, nanospray liquid chromatography-MS/MS was run in technical triplicate, and all measurements were performed at room temperature. Technical details of the mass spectrometry analyses can be found in the Additional Files (see Additional file 1). The threshold for peptide identification was set at 95% confidence and the stringency for protein identification was set at 99% confidence with at least 2 peptide matches.

### Data processing and analysis

Protein expression data was captured in the form of spectral counts, and any non-integer values were rounded up to the nearest whole integer. Each identified protein was mapped to a single gene symbol and Entrez ID. For protein isoforms, expression counts were summed to generate a single dataset for each gene. Such 1:1 mapping was required in downstream analyses. The R programming environment (version 3.2.1) [35] was used to process the spectral count data as described above, to perform statistical calculations, and to plot data. Differential protein expression between RC-77 T/E and RC-77 N/E cell lines was assessed using the processed spectral count data by an unpaired Wilcoxon rank-sum test with an applied continuity correction and two-sided alternative hypothesis via a built-in R function. Differentially expressed proteins (DEPs) were defined as those proteins whose mean spectral count differed between the two comparison sets with at least 90% confidence after adjusting for the false discovery rate using the Benjamini-Hochberg function. Next, fold changes in protein expression levels between RC-77 T/E and RC-77 N/E cell lines were calculated by taking the base 2 logarithm (log_2_) of the ratio of the mean spectral count of RC-77 T/E samples to the mean spectral count of RC-77 N/E samples. In this way, proteins downregulated in RC-77 T/E showed negative fold changes, whereas proteins upregulated in RC-77 T/E showed positive fold changes. For samples with zero means, the data was transformed by adding one to both means, which did not substantially affect the results of downstream analysis. A MA plot was constructed to confirm that variance remained stable (see Additional file 2).

### Overrepresentation analysis

To reveal any patterns in the classes or functions of proteins differentially expressed between RC-77 T/E and RC-77 N/E cell lines, DEPs were subjected to overrepresentation analysis using Protein ANalysis THrough Evolutionary Relationships (PANTHER) analysis tools [36]. The list of DEPs was loaded into the PANTHER Classification System data analysis tool (version 11.1), which sorted the DEPs by PANTHER protein class and Gene Ontology (GO) annotations. Using the same list of DEPs, the PANTHER statistical overrepresentation tool (release 20,161,024) was used to assess the probability that the number of DEPs belonging to each protein class or GO category was greater than the number expected in each category picked at random based on a reference human genome. Additionally, the overrepresentation of entire pathways among DEPs was assessed using the National Cancer Institute-Nature Pathway Interaction Database [37]. The list of DEPs was uploaded and searched against this database, and the overrepresentation of pathways was calculated, adjusting probabilities for multiple-hypotheses testing. To determine if the results obtained for DEPs were due to random chance, the same overrepresentation analyses were conducted for 1000 random sets containing the same number of proteins as DEPs sampled from the remaining non-differentially expressed proteins and from the total number of identified proteins detected by mass spectrometry.

### Gene set enrichment analysis

Gene Set Enrichment Analysis (GSEA) (version 2.2.0), which is a type of correlation analysis that uses expression data to associate gene sets with a particular phenotype [38], was used to identify groups of genes associated with either RC-77 T/E or RC-77 N/E cells. So as not to bias against small changes in expression, the processed protein spectral count data were inputted into the software without filtering for differential expression, and the log_2_ fold change was ignored. Proteins that could not be mapped to an Entrez ID were excluded from this analysis. Gene sets containing a minimum of 5 genes and up to a maximum of 500 genes were pulled from BioCarta and Reactome databases (downloaded from the GSEA’s Molecular Signatures Database, version 5) and from a customized database of relevant KEGG (Kyoto Encyclopedia of Genes and Genomes) pathways (see Additional file 3). The GSEA software interrogated each gene set against a list of the protein data ranked by correlation to RC-77 T/E or RC-77 N/E samples to determine which proteins from the ranked list appeared in a given pathway and whether they were randomly distributed or clustered among a phenotype. Enrichment (relative to RC-77 N/E) was based on the number of highly correlated genes from the ranked list that appeared in the pathway with a chosen FDR cut-off of q < 0.25.

### Signaling pathway impact analysis

Signaling Pathway Impact Analysis (SPIA) was used to provide a system-level assessment of pathway significance by incorporating overrepresentation, a function of differential expression and the magnitude of expression change (as a log_2_ ratio), and topology, the position of the protein in a pathway [39]. Pathway topology is important because it distinguishes genes or proteins that may be at trigger, regulatory, divergent, or end positions. SPIA was completed using the “SPIA” R package (version 2.18.0). The processed protein spectral count data including the results of the differential expression analysis and log_2_ fold changes were uploaded. Proteins that could not be mapped to an Entrez ID were excluded from this analysis. The threshold for differential expression was set to q < 0.1. The same relevant KEGG pathways used in GSEA were used for SPIA (see Additional file 3). KEGG pathways were chosen because they contain information about pathway topology. SPIA calculated the overrepresentation and perturbation probabilities and combined them into a global probability that a pathway was activated or inhibited in RC-77 T/E cells. The overrepresentation probability reflects the likelihood the number of DEPs observed in a pathway was larger than that observed by random chance. The perturbation probability reflects whether the positions of DEPs in a particular pathway were at crucial junctions that could perturb the pathway. The false discovery rate-adjusted global probability was the metric used to rank the significance of the pathways.

### Analysis of DEPs relevance in human prostate cancer patient specimens

Using The Cancer Genome Atlas (TCGA) prostate adenocarcinoma (PRAD) cohort, a dataset of 12 age- and stage-matched African-American and Caucasian-American specimen pairs (24 specimens total) was created. These specimen pairs were used to investigate how the protein and RNA expression of the 63 DEPs differed by race. To generate the dataset, TCGA protein data was downloaded from CBioportal, and TCGA RNA expression data was downloaded from FireBrowse.org. Both are repositories for TCGA data. The protein data available from the TCGA PRAD cohort was obtained via Reverse Phase Protein Array and was limited to 219 proteins. TCGA RNA expression data was obtained through Illumina HiSeq (RNA sequencing) and comprised over 20,000 gene transcripts. Only DEPs present in both datasets were carried forward for further analysis. Because the RC-77 T/E cell line was generated from an early stage primary tumor, only tumors with a Gleason score of 6 or 7 were included (see Additional file 4). Data frames of extracted protein and RNA expression data were created with Microsoft Excel.

Because protein data for non-malignant PRAD specimens was not available in TCGA data and non-malignant PRAD tissue was not collected from all patients, direct tumor-to-non-malignant comparisons could not be performed. In order to compare expression distributions, the average of the race-specific non-malignant PRAD RNA expression was subtracted from the age- and stage-matched tumor specimens (see Additional file 4). Of the 499 individuals in TCGA PRAD patient cohort, 51 had non-malignant PRAD tissue RNA expression data. After filtering for Gleason score (≤ 7), 34 (4 African-American and 30 Caucasian-American) non-malignant prostate tissue specimens were included in the non-malignant-expression-normalized analysis (see Additional file 4). The statistical significance of differences between African-American and Caucasian-American patient specimens were analyzed using the “t.test” function in R.

## Results

**Fig. 1:**
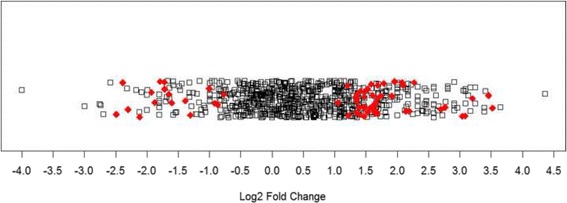
Magnitude of protein expression changes between RC-77 T/E and RC-77 N/E cell lines. In this one-dimensional scatter plot, the magnitude of protein expression changes is represented by log_2_ fold ratio. Red diamonds represent differentially expressed proteins. Black squares represent other identified proteins that were not significantly different

Overall, 843 proteins were identified by mass spectrometry, and 833 proteins remained in the dataset after processing to consolidate isoforms (see Additional files 5 and 6, respectively). These 833 proteins formed the dataset used in GSEA and SPIA analysis. Between RC-77 T/E and RC-77 N/E cell lines, 744 proteins were shared, 74 proteins were detected in RC-77 T/E cells but not RC-77 N/E cells, and 15 proteins were detected in RC-77 N/E but not RC-77 T/E cells. In total, expression levels of 200 proteins varied between RC-77 T/E and RC-77 N/E cells (*p* < 0.05, Wilcoxon rank-sum test); but after correcting for the false-discovery rate, only 63 proteins retained significance (q < 0.1). These 63 proteins formed the list of DEPs: 17 proteins downregulated in RC-77 T/E cells and 46 proteins upregulated in RC-77 T/E cells (Table 1). A full listing of protein expression changes between RC-77 N/E and RC-77 T/E cells is found in the Additional files (see Additional file 6). The distribution of log_2_ fold changes for all proteins was plotted in a 1-D scatter plot (Fig. 1). DEPs tended to have greater than two-fold changes in expression levels, and most log_2_ fold changes clustered around −2.0 and +1.5. The reproducibility among biological replicates was good (see Additional files 7 and 8).

**Table 1:** Differentially expressed proteins between RC-77 T/E and RC-77 N/E cell lines

Identified Proteins (Gene Symbol)	*p*-value	*q*-value	Log_2_ Fold Change	Status in RC-77 T/E Cells	Significant Pathway or Gene Set Involvement
CD166 antigen (ALCAM)	5.90E-04	4.91E-02	−2.12	Downregulated	
*Caveolin-1 (CAV1)	2.98E-04	4.91E-02	−1.72	Downregulated	Focal Adhesion; Proteoglycans in Cancer
*Vimentin (VIM)	4.09E-04	4.91E-02	−1.61	Downregulated	
*Myosin heavy chain-9 (MYH9)	4.04E-04	4.91E-02	1.58	Upregulated	
SH3 domain-binding glutamic acid-rich-like protein 3 (SH3BGRL3)	2.68E-04	4.91E-02	2.70	Upregulated	
Eukaryotic translation initiation factor 4B (EIF4B)	5.78E-04	4.91E-02	2.77	Upregulated	
Calpastatin (CAST)	3.55E-04	4.91E-02	3.09	Upregulated	
Nucleolar RNA helicase 2 (DDX21)	4.16E-04	4.91E-02	3.20	Upregulated	
Creatine kinase U-type (CKMT1A)	3.36E-04	4.91E-02	3.46	Upregulated	
Thioredoxin domain-containing protein 17 (TXNDC17)	4.92E-04	4.91E-02	1.69	RC-77 T/E only	
*Type I cytoskeletal keratin 19 (KRT19)	7.77E-04	5.40E-02	−2.49	Downregulated	
Serotransferrin (TF)	7.29E-04	5.40E-02	−2.30	Downregulated	
Integrin alpha-6 (ITGA6)	1.44E-03	5.40E-02	−1.93	Downregulated	Cell Adhesion Molecules; ECM-Receptor Interaction; Small Cell Lung Cancer
Laminin subunit gamma-2 (LAMC2)	9.86E-04	5.40E-02	−1.72	Downregulated	ECM-Receptor Interaction; Small Cell Lung Cancer; Focal Adhesion
CD59 glycoprotein (CD59)	9.15E-04	5.40E-02	−1.65	Downregulated	
Squalene synthase (FDFT1)	1.23E-03	5.40E-02	−1.31	Downregulated	
*Filamin-A (FLNA)	1.06E-03	5.40E-02	1.21	Upregulated	Focal Adhesion, Proteoglycans in Cancer
Hydroxyacyl-coenzyme A dehydrogenase (HADH)	1.61E-03	5.40E-02	1.22	Upregulated	
X-ray repair cross-complementing protein 5 (XRCC5)	1.42E-03	5.40E-02	1.35	Upregulated	
Prothymosin alpha (PTMA)	1.49E-03	5.40E-02	1.65	Upregulated	
Cytosolic acyl coenzyme A thioester hydrolase (ACOT7)	1.37E-03	5.40E-02	1.74	Upregulated	
High mobility group protein HMG-I/HMG-Y (HMGA1)	1.58E-03	5.40E-02	1.79	Upregulated	
Putative pre-mRNA-splicing factor ATP-dependent RNA helicase DHX15 (DHX15)	1.10E-03	5.40E-02	2.10	Upregulated	
Scaffold attachment factor B1 (SAFB)	1.59E-03	5.40E-02	2.27	Upregulated	
Nucleoprotein TPR (TPR)	1.62E-03	5.40E-02	3.52	Upregulated	
Hemoglobin subunit alpha (HBA1)	1.80E-03	5.54E-02	−1.87	RC-77 N/E only	
Protein PML (PML)	1.77E-03	5.54E-02	1.69	RC-77 T/E only	
Ribosome-binding protein 1 (RRBP1)	1.89E-03	5.63E-02	1.64	Upregulated	
Adenosylhomocysteinase (AHCY)	2.00E-03	5.75E-02	1.68	Upregulated	
Gamma-interferon-inducible protein 16 (IFI16)	2.37E-03	6.59E-02	1.39	Upregulated	
Phosphoenolpyruvate carboxykinase (PCK2)	2.51E-03	6.75E-02	3.04	Upregulated	
14–3-3 protein sigma (SFN)	2.60E-03	6.76E-02	1.49	Upregulated	
*Lamin-B1 (LMNB1)	3.04E-03	7.26E-02	−0.87	Downregulated	
*Alpha-actinin-1 (ACTN1)	3.05E-03	7.26E-02	1.06	Upregulated	Tight Junction; Adherens Junction; Hippo Signaling Pathway; Focal Adhesion
High mobility group protein HMGI-C (HMGA2)	2.95E-03	7.26E-02	2.19	Upregulated	
Voltage-dependent anion-selective channel protein 1 (VDAC1)	4.59E-03	7.67E-02	−1.00	Downregulated	
Integrin beta-1 (ITGB1)	3.48E-03	7.67E-02	−0.92	Downregulated	Cell Adhesion Molecules; ECM-Receptor Interaction; Small Cell Lung Cancer
Non-histone chromosomal protein HMG-17 (HMGN2)	4.22E-03	7.67E-02	1.34	Upregulated	
*PDZ and LIM domain protein 1 (PDLIM1)	4.40E-03	7.67E-02	1.61	Upregulated	
T-complex protein 1 subunit epsilon (CCT5)	4.72E-03	7.67E-02	1.66	Upregulated	
Aminopeptidase N (ANPEP)	5.25E-03	7.67E-02	−2.38	RC-77 N/E only	
Prefoldin subunit 2 (PFDN2)	4.96E-03	7.67E-02	1.35	RC-77 T/E only	
40S ribosomal protein S24 (RPS24)	4.96E-03	7.67E-02	1.35	RC-77 T/E only	
Serine/arginine-rich splicing factor 1 (SRSF1)	4.96E-03	7.67E-02	1.35	RC-77 T/E only	
S-formylglutathione hydrolase (ESD)	5.05E-03	7.67E-02	1.42	RC-77 T/E only	
RNA-binding protein EWS (EWSR1)	5.15E-03	7.67E-02	1.47	RC-77 T/E only	
Hepatoma-derived growth factor (HDGF)	5.15E-03	7.67E-02	1.47	RC-77 T/E only	
Non-histone chromosomal protein HMG-14 (HMGN1)	4.96E-03	7.67E-02	1.47	RC-77 T/E only	
S-methyl-5′-thioadenosine phosphorylase (MTAP)	4.96E-03	7.67E-02	1.47	RC-77 T/E only	
Phosphoserine aminotransferase (PSAT1)	5.15E-03	7.67E-02	1.53	RC-77 T/E only	
60S ribosomal protein L10 (RPL10)	4.99E-03	7.67E-02	1.53	RC-77 T/E only	
Proteasome activator complex subunit 3 (PSME3)	5.22E-03	7.67E-02	1.58	RC-77 T/E only	
40S ribosomal protein S11 (RPS11)	4.99E-03	7.67E-02	1.64	RC-77 T/E only	
tRNA-splicing ligase RtcB homolog (RTCB)	5.25E-03	7.67E-02	1.64	RC-77 T/E only	
Double-stranded RNA-specific adenosine deaminase (ADAR)	5.25E-03	7.67E-02	1.92	RC-77 T/E only	
Eukaryotic translation initiation factor 3 subunit I (EIF3I)	5.22E-03	7.67E-02	1.96	RC-77 T/E only	
60S ribosomal protein L35 (RPL35)	5.18E-03	7.67E-02	2.08	RC-77 T/E only	
Cytochrome c oxidase subunit 5A (COX5A)	5.74E-03	8.24E-02	−1.38	Downregulated	
*Beta-catenin (CTNNB1)	5.93E-03	8.37E-02	1.40	Upregulated	Tight Junction; Adherens Junction; Hippo Signaling Pathway; Focal Adhesion
*Type II cytoskeletal keratin 8 (KRT8)	6.14E-03	8.52E-02	−1.79	Downregulated	
CD44 antigen (CD44)	6.44E-03	8.60E-02	−0.77	Downregulated	Proteoglycans in Cancer; ECM-Receptor Interaction
Plasminogen activator inhibitor 1 RNA-binding protein (SERBP1)	6.51E-03	8.60E-02	1.58	Upregulated	
60S ribosomal protein L6 (RPL6)	6.38E-03	8.60E-02	2.14	Upregulated	

*Carries a “Structural” or “Cytoskeletal” annotation in PANTHER. *P*-value is the probability the protein differs between RC-77 N/E and RC-77 T/E as calculated by an unpaired Wilcoxon rank-sum test, and q-value is the probability adjusted for multiple hypotheses testing using the Benjamini-Hochberg method. The log_2_ fold change was calculated using the RC-77 T/E to RC-77 N/E ratio. Significant pathway or gene set involvement reflects the results of Gene Set Enrichment Analysis and Signaling Pathway Impact Analysis

### Overrepresentation analysis

**Fig. 2:**
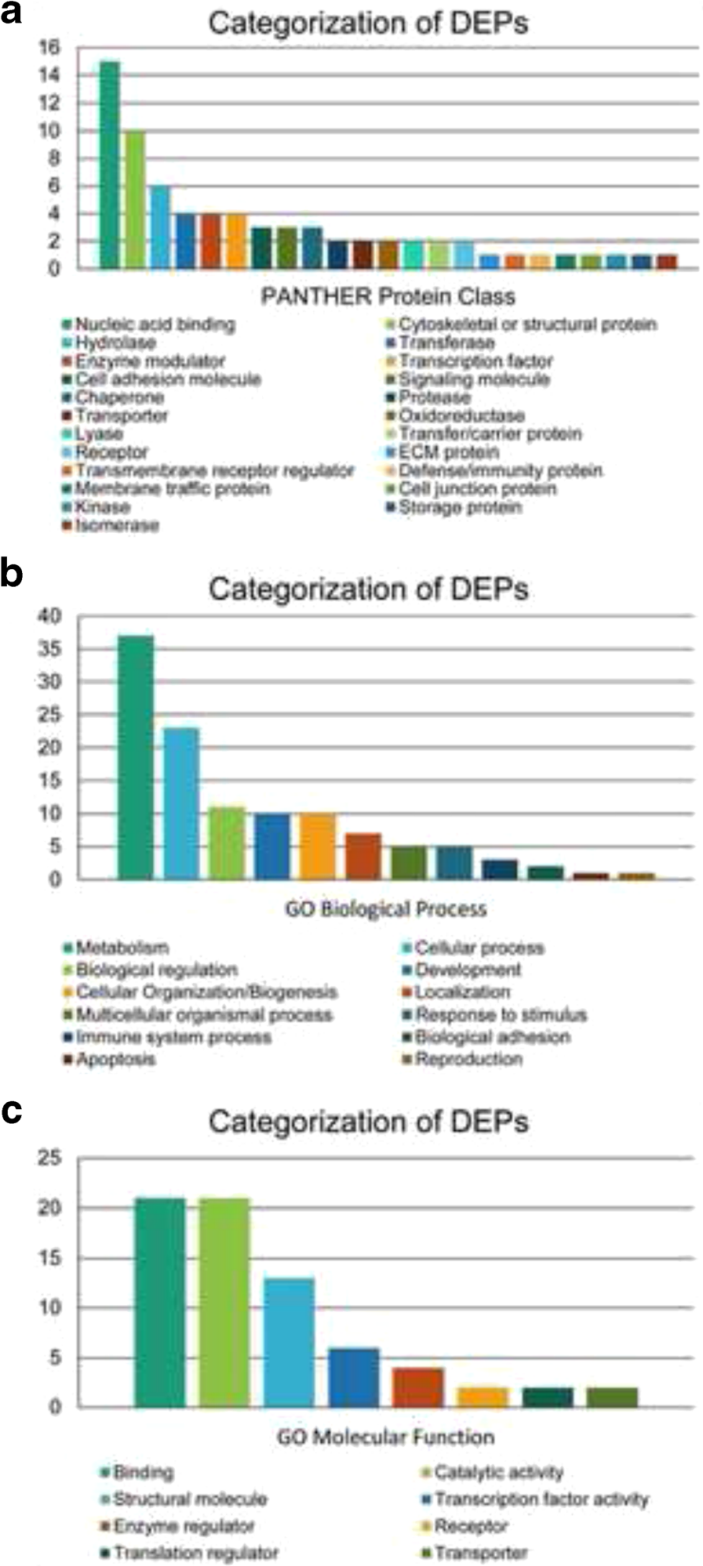
Functional classification of differentially expressed proteins between RC-77 T/E and RC-77 N/E cell lines. DEPs in RC-77 T/E and RC-77 N/E cell lines were classified according to (A) PANTHER protein class, (B) Biological Process Gene Ontology terms, and (C) Molecular Function Gene Ontology terms. Note: No annotations were found for 12 DEPs (laminin subunit gamma-2, SH3 domain-binding glutamic acid-rich-like protein 3, serine/arginine-rich splicing factor 1, CD44 antigen, tRNA-splicing ligase RtcB homolog, ribosome-binding protein 1, scaffold attachment factor B1, nucleoprotein TPR, integrin alpha-6, protein PML, squalene synthase, and X-ray repair cross-complementing protein 5). DEP = differentially expressed protein; PANTHER = PANTHER: Protein ANalysis THrough Evolutionary Relationships

For each of the 63 DEPs, PANTHER protein class and GO annotations were pulled from the PANTHER database, and the number of annotations in each category were counted (Fig. 2). No annotations were found for 12 DEPs; however, a pattern of nucleic acid binding and structural proteins emerged among the annotations for the 51 remaining DEPs. “Nucleic Acid Binding” was the most populated PANTHER protein class category with 15 DEPs, while 10 DEPs were classified as “Structural” and/or “Cytoskeletal Proteins”, and another 6 DEPs were classified as hydrolases (Table 2). The remaining DEPs were spread nearly evenly across 20 other categories (Fig. 2a). When DEPs were sorted by GO Molecular Function notation (Fig. 2c), the “Binding” and “Catalytic Activity” GO Molecular Function labels each covered over 40% (21 of 51 DEPs) of the annotated DEPs, and the “Structural Molecule Activity” label was also highly populated (13 of 51 DEPs) (Table 3). Overrepresentation analysis supported the pattern of structural/cytoskeletal proteins among proteins differentially expressed between RC-77 T/E and RC-77 N/E cells (Table 4). Only the “Cytoskeletal Protein” PANTHER protein class category (q = 0.033) was statistically overrepresented among the DEPs compared to the reference human genome/proteome (20,814 genes/proteins).

**Table 2:** Categorization of differentially expressed proteins according to PANTHER protein class

PANTHER Protein Class (Number of Differentially Expressed Proteins)		
Nucleic Acid Binding Proteins (15)		
• eukaryotic translation initiation factor 4B	• RNA-binding protein EWS	• high mobility group protein HMG-I/HMG-Y
• high mobility group protein HMGI-C	• non-histone chromosomal protein HMG-14	• non-histone chromosomal protein HMG-17
• 40S ribosomal protein S24	• nucleolar RNA helicase 2	• 60S ribosomal protein L35
• 60S ribosomal protein L6	• 40S ribosomal protein S11	• 60S ribosomal protein L10
• plasminogen activator inhibitor 1 RNA-binding protein	• putative pre-mRNA-splicing factor ATP-dependent RNA helicase DHX15	• double-stranded RNA-specific adenosine deaminase
Structural and/or Cytoskeletal Proteins (10)		
• beta-catenin	• filamin-A	• vimentin
• lamin-B1	• alpha-actinin-1	• caveolin-1
• PDZ and LIM domain protein 1	• type I cytoskeletal keratin 19	• type II cytoskeletal keratin 8
• myosin heavy chain-9		
Hydrolases (6)		
• serotransferrin	• aminopeptidase N	• adenosylhomocysteinase
• double-stranded RNA-specific adenosine deaminase	• cytosolic acyl coenzyme A thioester hydrolase	• S-formylglutathione hydrolase

**Table 3:** Categorization of differentially expressed proteins according to Gene Ontology Molecular Function

Gene Ontology Molecular Function Annotation (Number of Differentially Expressed Proteins)		
Binding Proteins (21)		
• eukaryotic translation initiation factor 4B	• RNA-binding protein EWS	• high mobility group protein HMG-I/HMG-Y
• double-stranded RNA-specific adenosine deaminase	• plasminogen activator inhibitor 1 RNA-binding protein	• non-histone chromosomal protein HMG-17
• alpha-actinin-1	• nucleolar RNA helicase 2	• 60S ribosomal protein L35
• hepatoma-derived growth factor	• gamma-interferon-inducible protein 16	• 60S ribosomal protein L10
• PDZ and LIM domain protein 1	• non-histone chromosomal protein HMG-14	• high mobility group protein HMGI-C
• caveolin-1	• calpastatin	• beta-catenin
• 60S ribosomal protein L6	• filamin-A	• myosin heavy chain-9
Catalytic Activity Proteins (21)		
• serotransferrin	• aminopeptidase N	• calpastatin
• double-stranded RNA-specific adenosine deaminase	• putative pre-mRNA-splicing factor ATP-dependent RNA helicase DHX15	• type I cytoskeletal high mobility group protein HMG-I/HMG-Y
• cytosolic acyl coenzyme A thioester hydrolase	• hydroxyacyl-coenzyme A dehydrogenase	• S-formylglutathione hydrolase
• phosphoenolpyruvate carboxykinase	• cytochrome c oxidase subunit 5A	• S-methyl-5′-thioadenosine phosphorylase
• creatine kinase U-type	• 60S ribosomal protein L35	• caveolin-1
• myosin heavy chain-9	• adenosylhomocysteinase	• nucleolar RNA helicase 2
• high mobility group protein HMGI-C	• phosphoserine aminotransferase	• RNA-binding protein EWS
Structural Molecule Activity (13)		
• filamin-A	• 60S ribosomal protein L10	• 60S ribosomal protein L35
• Type I cytoskeletal keratin 19	• type II cytoskeletal keratin 8	• PDZ and LIM domain protein 1
• Myosin heavy chain-9	• 60S ribosomal protein L6	• 40S ribosomal protein S11
• alpha-actinin-1	• vimentin	• lamin-B1
• caveolin-1

**Table 4:** Overrepresentation analysis by PANTHER protein class of differentially expressed proteins and random sets of proteins

Protein Class	Size of Class	Overrepresentation Analysis		# Sets per 1000 in Which Significantly Overrepresented	
		*p*-value	*q*-value	Using non-DEPs	Using all Proteins
cytoskeletal protein	198	0.001	0.033	2	0
storage protein	25	0.011	0.123	0	7
chaperone	183	0.027	0.209	100	107
transmembrane receptor regulatory	65	0.062	0.312	0	1
lyase	151	0.068	0.312	28	33
nucleic acid binding	2332	0.182	0.697	1	7
cell junction protein	140	0.216	0.71	0	0
isomerase	162	0.266	0.766	21	16
cell adhesion molecule	458	0.317	0.81	0	0
extracellular matrix protein	363	0.393	0.825	0	0
transfer/carrier protein	364	0.395	0.825	3	3
protease	586	0.497	0.953	0	0
membrane traffic protein	372	0.677	0.992	1	1
oxidoreductase	593	0.72	0.992	94	61
kinase	699	0.818	0.992	0	0
hydrolase	1482	0.832	0.992	0	0
defense/immunity protein	561	0.87	0.992	0	0
transferase	1198	0.879	0.992	0	0
signaling molecule	1083	0.915	0.992	0	0
transporter	920	0.933	0.992	0	0
enzyme modulator	1353	0.935	0.992	0	0
transcription factor	1451	0.957	0.992	0	0
receptor	1813	0.992	0.992	0	0

The PANTHER overrepresentation analysis was run on the subset of 63 DEPs and on 1000 subsets of 63 proteins (the number of DEPs identified) randomly sampled from the 770 non-differentially expressed proteins and from all 833 proteins identified by mass spectrometry. Overrepresentation was based on comparison to the reference human genome/proteome. *DEP* differentially expressed protein, *PANTHER* PANTHER: Protein ANalysis THrough Evolutionary Relationships

Because structural and cytoskeletal proteins are highly abundant, we verified the results of the enrichment and overrepresentation of this protein class by comparing the results to those obtained using an equivalent number of randomly sampled proteins. We repeated the overrepresentation analysis on 1000 subsets of 63 proteins (the number of DEPs identified) randomly sampled from the 770 non-differentially expressed proteins and from all 833 proteins identified by mass spectrometry compared to the reference human genome/proteome. Among the repeated sets of proteins pulled from the 770 non-DEPs, structural/cytoskeletal proteins protein were significantly overrepresented in only 2 sets; there were no sets from the proteins sampled from all 833 proteins with significant overrepresentation of the structural/cytoskeletal protein class (Table 4). Therefore, we conclude with high probability (99.8%) that the overrepresentation of the structural/cytoskeletal protein class among the 63 DEPs is not by random chance. In contrast, many DEPs were labeled with the “Catalytic Activity” GO Molecular Function; however, enzyme protein classes were not overrepresented according to the enrichment test and were more frequent among the random samples. These results verified that the differences between RC-77 T/E and RC-77 N/E cell lines are specifically linked to structural/cytoskeletal proteins because none of the 1000 random subsets of proteins from 770 non-DEPs were enriched in structural proteins relative to the genome/proteome.

There was a deviation from the pattern of structural/cytoskeletal protein overrepresentation when DEPs were analyzed by GO Biological Process annotations. Metabolic and cellular processes were the most common GO Biological Process annotation, with 37 and 23 proteins, respectively (Fig. 2B and Table 5). The GO Biological Process category “Metabolic Process” encompasses carbohydrate, lipid, protein, amino acid, and nucloeobase-containing compound metabolism; and the GO Biological Process term “Cellular Process” is an umbrella heading for cell communication, cell cycle, cytokinesis, and cellular component movement. The GO Biological Process categories “Biological Regulation”, “Developmental Process”, and “Cellular Component Organization or Biogenesis” were evenly populated (Fig. 2b).

**Table 5:** Categorization of differentially expressed proteins according to Gene Ontology Biological Process

Gene Ontology Biological Process Annotation (Number of Differentially Expressed Proteins)		
Metabolic Process (37)		
• eukaryotic translation initiation factor 4B	• double-stranded RNA-specific adenosine deaminase	• plasminogen activator inhibitor 1 RNA-binding protein
• serotransferrin	• 60S ribosomal protein L6	• nucleolar RNA helicase 2
• proteasome activator complex subunit 3	• non-histone chromosomal protein HMG-14	• cytosolic acyl coenzyme A thioester hydrolase
• phosphoenolpyruvate carboxykinase	• non-histone chromosomal protein HMG-17	• cytochrome c oxidase subunit 5A
• prothymosin alpha	• 40S ribosomal protein S11	• 40S ribosomal protein S24
• high mobility group protein HMG-I/HMG-Y	• gamma-interferon-inducible protein 16	• T-complex protein 1 subunit epsilon
• RNA-binding protein EWS	• PDZ and LIM domain protein 1	• high mobility group protein HMGI-C
• calpastatin	• 60S ribosomal protein L35	• aminopeptidase N
• creatine kinase U-type	• myosin heavy chain-9	• 60S ribosomal protein L10
• S-formylglutathione hydrolase	• hepatoma-derived growth factor	• phosphoserine aminotransferase
• thioredoxin domain-containing protein 17	• S-methyl-5′-thioadenosine phosphorylase	• hydroxyacyl-coenzyme A dehydrogenase
• adenosylhomocysteinase	• prefoldin subunit 2	• caveolin-1
• putative pre-mRNA- splicing factor ATP-dependent RNA helicase DHX15		
Cellular Process Proteins (23)		
• PDZ and LIM domain protein 1	• type I cytoskeletal keratin 19	• non-histone chromosomal protein HMG-17
• lamin-B1	• integrin beta-1	• CD166 antigen
• non-histone chromosomal protein HMG-14	• double-stranded RNA-specific adenosine deaminase	• high mobility group protein HMG-I/HMG-Y
• myosin heavy chain-9	• 40S ribosomal protein S11	• caveolin-1
• vimentin	• 40S ribosomal protein S24	• filamin-A
• high mobility group protein HMGI-C	• type II cytoskeletal keratin 8	• hepatoma-derived growth factor
• CD59 glycoprotein	• alpha-actinin-1	• 14–3-3 protein sigma
• adenosylhomocysteinase	• putative pre-mRNA-splicing factor ATP-dependent RNA helicase DHX15	

In addition to grouping by PANTHER protein class or GO annotations, pathway overrepresentation among the DEPs was also assessed using the National Cancer Institute-Nature Pathway Interaction Database. Again, structural molecules featured prominently in these pathways, including integrin alpha-6, integrin beta-1, and beta-catenin (Table 6).

**Table 6:** Pathways from the National Cancer Institute-Nature Pathway Interaction Database overrepresented in RC-77 cell lines

Pathway Name	Differentially Expressed Proteins in Pathway	*p*-value	*q*-value
α6β1 and α6β4 integrin signaling	*ITGA6*, *ITGB1*, *LAMC2*, **SFN**	5.75E-05	7.88E-03
α4β7 integrin signaling	*CD44*, *ITGB1*	7.79E-04	5.14E-02
α6β4 integrin-ligand interactions	*ITGA6*, *LAMC2*	1.18E-03	5.14E-02
Arf6 trafficking events	**CTNNB1**, *ITGA6*, *ITGB1*	1.50E-03	5.14E-02
TGF-beta receptor signaling	*CAV1*, **CTNNB1**, PML*	2.08E-03	5.70E-02
mTOR signaling pathway	**EIF4B**, PML*, **SFN**	4.07E-03	7.38E-02
β1 integrin cell surface interactions	*ITGA6*, *ITGB1*, *LAMC2*	4.07E-03	7.38E-02
Canonical Wnt signaling pathway	*CAV1*, **CTNNB1**	4.31E-03	7.38E-02
Plexin-D1 signaling	*ITGA6*, *ITGB1*	5.59E-03	8.51E-02
Integrin family cell surface interactions	*ITGA6*, *ITGB1*	6.52E-03	8.93E-02
BARD1 signaling events	EWSR1*, **XRCC5**	8.58E-03	1.07E-01
Syndecan-4-mediated signaling events	**ACTN1**, *ITGB1*	9.69E-03	1.11E-01
Signaling mediated by p38-alpha and p38-beta	*KRT19*, *KRT8*	1.34E-02	1.40E-01
E-cadherin signaling events	**CTNNB1**	1.43E-02	1.40E-01
Stabilization and expansion of the E-cadherin adherens junction	**ACTN1**, **CTNNB1**	1.75E-02	1.60E-01
Integrin-linked kinase signaling	**ACTN1**, **CTNNB1**	1.90E-02	1.63E-01
FoxO family signaling	**CTNNB1**, **SFN**	2.21E-02	1.78E-01
Direct p53 effectors	*CAV1*, PML*, **SFN**	2.46E-02	1.87E-01
Caspase cascade in apoptosis	*LMNB1*, *VIM*	2.79E-02	2.01E-01
Co-regulation of androgen receptor activity	**CTNNB1**, **XRCC5**	3.32E-02	2.17E-01
Signaling events mediated by focal adhesion kinase	**ACTN1**, *ITGB1*	3.32E-02	2.17E-01
Validated targets of C-MYC transcriptional repression	*ITGA6*, *ITGB1*	4.27E-02	2.54E-01
Signaling events mediated by VEGFR1 and VEGFR2	*CAV1*, **CTNNB1**	4.27E-02	2.54E-01
p73 transcription factor network	PML*, **SFN**	4.88E-02	2.79E-01

Proteins in bold font were upregulated in RC-77 T/E. Proteins in italic font were downregulated in RC-77 T/E. *Protein found in RC-77 T/E only. *P*-values were calculated using a hypergeometric cumulative distribution function. Q-value is the *p*-value corrected for multiple hypotheses testing using the Benjamini-Hochberg method. *ACTN1* alpha-actinin-1, *CAV1* caveolin-1, *CD44* CD44 antigen, *CTNNB1* beta-catenin, *EIF4B* eukaryotic translation initiation factor 4B, *EWSR1* RNA-binding protein EWS, *ITGA6* integrin alpha-6, *ITGB1* integrin beta-1, *KRT8* type II cytoskeletal keratin 8, *KRT19* = type I cytoskeletal keratin 19, *LAMC2* laminin subunit gamma-2, *LMNB1* lamin-B1, *PML* protein PML, *SFN* 14–3-3 protein sigma, *VIM* vimentin, *XRCC5* X-ray repair cross-complementing protein 5

### Gene set enrichment analysis

Although overrepresentation analysis showed that structural proteins and pathways related to structural proteins differed between RC-77 T/E and RC-77 N/E cells, this analysis did not link these differences directly to either of the cell lines. GSEA identified groups of genes specifically associated with either RC-77 T/E or RC-77 N/E cells. For this analysis, all protein data were used as the input, not just data for the 63 DEPs. Multiple gene sets were enriched in RC-77 T/E and RC-77 N/E cells (Table 7). A complete listing of GSEA results is presented in the Additional files (see Additional file 9). An enriched gene set contained a significant number of proteins whose expression most correlated with either RC-77 T/E or RC-77 N/E cells. The most significantly enriched gene set in RC-77 T/E cells was the KEGG “Tight Junction” gene set. Additionally, the KEGG “Adherens Junction” gene set was highly enriched in RC-77 T/E cells. The most significant gene set enriched in RC-77 N/E cells was the KEGG “Cell Adhesion Molecules”, and the KEGG “ECM-Receptor Interaction” gene set was also highly enriched in RC-77 N/E cells. Interestingly, structural proteins contributed to the enrichment of each of these gene sets in their respective cell lines. While alpha-actinin-1 and beta-catenin were associated with RC-77 T/E cells, integrin alpha-6, integrin beta-1, laminin subunit gamma-2, and CD166 antigen were associated with RC-77 N/E cells. These results corroborate the overrepresentation of structural proteins in these cell lines. Furthermore, this enrichment analysis differentiates which structural protein was associated with each cell line.

**Table 7:** Enriched gene sets in RC-77 T/E and RC-77 N/E cell lines

Pathway (Size)	NES	*p*-value	*q*-value	Proteins Contributing to Enrichment
KEGG: Tight Junction (16)	2.064	0.00E + 00	3.41E-03	alpha-actinin-1*, alpha-actinin-4*, alpha-catenin, beta-catenin*, casein kinase II subunit beta, myosin heavy chain-9*, Src substrate cortactin*
KEGG: Cell Adhesion Molecules (6)	−1.836	0.00E + 00	1.34E-02	**CD166 antigen, integrin alpha-6, integrin beta-1**
KEGG: Hippo Signaling Pathway (9)	1.797	3.98E-03	2.73E-02	alpha-catenin*, beta-catenin*, 14–3-3 protein beta/alpha, 14–3-3 protein theta, 14–3-3 protein zeta/delta
KEGG: Transcriptional Misregulation in Cancer (9)	1.733	1.54E-02	4.09E-02	**high mobility group protein HMGI-C**, junction plakoglobin*, **protein PML**, **RNA-binding protein EWS**, RNA-binding protein FUS
KEGG: Adherens Junction (12)	1.704	1.27E-02	4.87E-02	alpha-actinin-1*, alpha-actinin-4*, alpha-catenin*, beta-catenin*, epidermal growth factor receptor, casein kinase II subunit beta
BioCarta: ChREBP2 Pathway (7)	1.695	1.01E-02	8.11E-02	14–3-3 protein beta/alpha, 14–3-3 protein theta, 14–3-3 protein zeta/delta, fatty acid synthase
KEGG: Cell Cycle (9)	1.627	2.49E-02	9.07E-02	DNA-dependent protein kinase catalytic subunit, DNA replication licensing factor MCM6, 14–3-3 protein beta/alpha, **14–3-3 protein sigma**, 14–3-3 protein theta, 14–3-3 protein zeta/delta,
KEGG: ECM-Receptor Interaction (8)	−1.557	6.09E-03	1.57E-01	**CD44 antigen**, integrin alpha-2, integrin alpha-3, **integrin alpha-6**, **integrin beta-1**, integrin beta-4, laminin subunit beta-3, **laminin subunit gamma-2**
KEGG: Small Cell Lung Cancer (7)	−1.488	5.20E-02	1.87E-01	integrin alpha-2, integrin alpha-3, **integrin alpha-6**, **integrin beta-1**, laminin subunit beta-3, **laminin subunit gamma-2**
KEGG: Complement and Coagulation Cascades (5)	−1.441	5.08E-02	2.02E-01	alpha-1-antitrypsin, alpha-2-macroglobulin, complement C3, **CD59 glycoprotein**, tissue factor

Positive enrichment scores correspond to enrichment in RC-77 T/E samples. Negative enrichment scores correspond to enrichment in RC-77 N/E samples. Bolded proteins were differentially expressed (q < 0.1, Wilcoxon rank-sum test). *Carries a “Structural” or “Cytoskeletal” annotation in PANTHER. *ChREBP2* carbohydrate responsive element binding protein, *ECM* extracellular matrix, *KEGG* Kyoto Encyclopedia of Genes and Genomes, *NES* normalized enrichment score (normalized to size of the pathway); *p*-value = probability of significance after permutation, q-value = false discovery rate-adjusted *p*-value; size = total number of genes in pathway

### Signaling pathway impact analysis

SPIA was conducted to address both the overrepresentation and pathway topology of DEPs to determine whether the DEPs found in a pathway have a meaningful impact within that pathway. SPIA differs from GSEA in two key ways. First, it considers the magnitude of expression and establishes a difference in impact between small and large fold changes. Second, by including a measure of perturbation, SPIA more fully captures the interactions of proteins, which can be lost in overrepresentation analyses and correlation analyses like GSEA. Four KEGG pathways were significantly impacted in the RC-77 T/E cell line: “Focal Adhesion” (false discovery rate-adjusted global probability [pGFdr] = 0.00934), “Small Cell Lung Cancer” (pGFdr = 0.0246), “Proteoglycans in Cancer” (pGFdr = 0.0246), and “ECM-Receptor Interaction” (pGFdr = 0.0246) (Table 8). Based on the expression pattern of the DEPs found in the pathway, SPIA predicted these four pathways were inhibited in RC-77 T/E cells. In corroboration, “ECM-Receptor Interaction” and “Small Cell Lung Cancer” were enriched in RC-77 N/E cells according to GSEA results. Pathway images with DEPs highlighted can be found in the full SPIA results presented in the Additional files (see Additional file 10). Note that not all components of the significantly impacted pathways were differentially expressed.

**Table 8:** Significantly inhibited pathways in RC-77 T/E cell lines

Name (KEGG ID)	NDE	pNDE	pPERT	pG	pGFdr
Focal Adhesion (hsa04510)	7	9.61E-04	2.00E-02	2.28E-04	9.34E-03
Small Cell Lung Cancer (hsa05222)	3	1.17E-02	1.40E-02	1.59E-03	2.46E-02
Proteoglycans in Cancer (hsa05205)	6	1.35E-03	1.78E-01	2.25E-03	2.46E-02
ECM-Receptor Interaction (hsa04512)	4	1.67E-03	1.55E-01	2.40E-03	2.46E-02

*ECM* extracellular matrix, *NDE* number of differentially expressed elements, *pG* global probability, *pGFdr* false discovery rate-adjusted global probability, *pNDE* overrepresentation probability, *pPERT*, perturbation probability

### Differentially expressed proteins with recurring pathway involvement

**Fig. 3:**
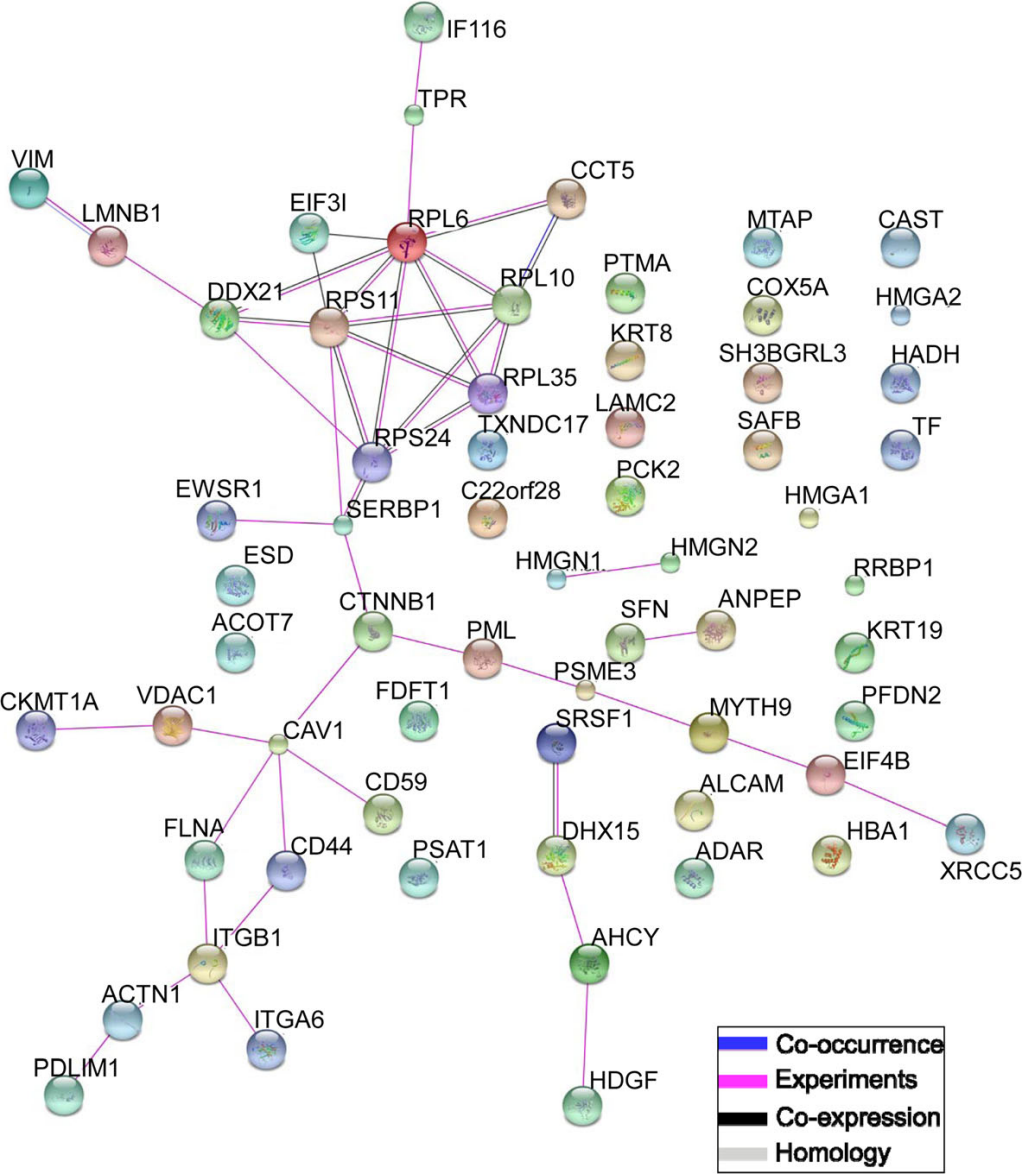
Functional associations between differentially expressed proteins in RC-77 T/E and RC-77 N/E cell lines. STRING (Search Tool for the Retrieval of Interacting Genes/Proteins) was used to visualize a network of functional associations between differentially expressed proteins. Interactions were limited to only those supported by experimental evidence, co-expression or co-occurrence data, and gene homology data. See Table 1 for the full names of proteins abbreviated here. Nodes centered on integrin beta-1, beta-catenin, and caveolin-1, suggesting these proteins have the potential to affect other proteins and may be involved in functional networks

Many of the significant pathways featured a small recurring group of DEPs: beta-catenin, alpha-actinin-1, integrin beta-1, integrin alpha-6, caveolin-1, filamin-A, laminin subunit gamma-2, and CD44 antigen (Table 1). Beta-catenin and alpha-actinin-1 contributed to the significance of the “Tight Junction”, “Adherens Junction”, “Hippo Signaling Pathway”, and “Focal Adhesion” pathways. Integrin beta-1 and integrin alpha-6 were included in the “Cell Adhesion Molecules”, “Small Cell Lung Cancer”, and “ECM-Receptor Interaction” pathways. Caveolin-1 and filamin A were included in the “Focal Adhesion” and “Proteoglycans in Cancer” pathways. Laminin subunit gamma-2 appeared in the “ECM-Receptor Interaction”, “Small Cell Lung Cancer”, and “Focal Adhesion” pathways. Finally, CD44 antigen appeared in the “Proteoglycans in Cancer” and “ECM-Receptor Interaction”. Experimental, co-expression, co-occurrence, and homology interactions between DEPs were visualized using STRING (Search Tool for the Retrieval of Interacting Genes/Proteins) [40] (Fig. 3). This plot displays direct interactions between DEPs. Nodes were centered on integrin beta-1, beta-catenin, and caveolin-1, suggesting these proteins have the potential to affect other proteins and may be involved in functional networks.

### Differentially expressed proteins and genes in human prostate cancer patient specimens

**Fig. 4:**
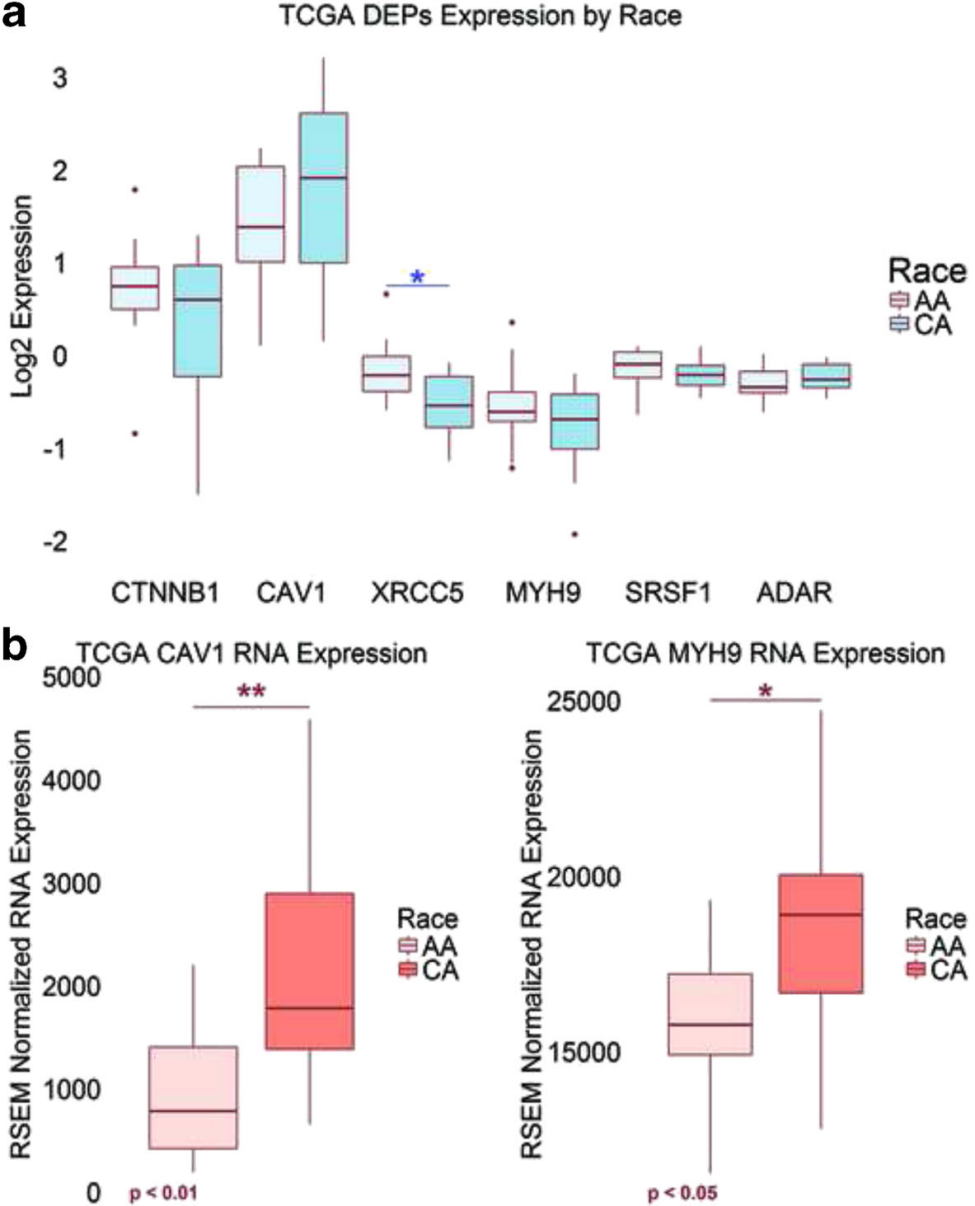
Expression of differentially expressed proteins by race in age- and stage-matched human prostate cancer specimens. In 12 age-and stage-matched prostate cancer specimen pairs extracted from TCGA without subtracting the non-malignant controls, (A) XRCC5 protein was found to be significantly different (*p* < 0.05) between African-American and Caucasian-American prostate cancer specimens and (B) RNA expression of CAV1 and MYH9 were found to be significantly different (*p* < 0.01 and <0.05, respectively) between African-American and Caucasian-American prostate cancer specimens. The *p*-values were generated using the “t.test” function in R. AA = African-American; ADAR = double-stranded RNA-specific adenosine deaminase; CA = Caucasian-American; CAV1 = caveolin-1; CTNNB1 = beta-catenin; MYH9 = myosin heavy chain-9; SRSF1 = serine/arginine-rich splicing factor 1; TCGA = The Cancer Genome Atlas; XRCC5 = X-ray repair cross-complementing protein 5

**Fig. 5:**
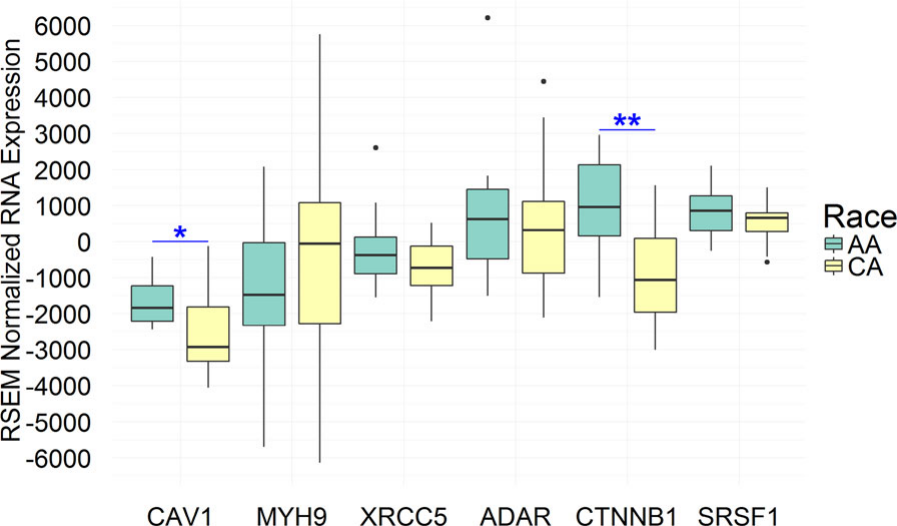
Tumor-to-non-malignant comparison of RNA expression of the differentially expressed proteins by race in age- and stage-matched human prostate cancer specimens. Race-specific non-malignant mRNA expression levels of PRAD specimens were subtracted from 12 pairs of age- and stage-matched prostate cancer specimens extracted from TCGA, respectively. CAV1 and CTNNB1 mRNA expressions were found to be significantly higher in African-American compared to Caucasian-American specimens (*p* < 0.05 and <0.01, respectively). The *p*-values were generated using the “t.test” function in R. As indicated by the negative RNA expression value on the y-axis, CAV1 was downregulated in African American prostate cancer specimens compared to African American non-malignant control specimens. On the contrary, CTNNB1 was upregulated. AA = African-American; ADAR = double-stranded RNA-specific adenosine deaminase; CA = Caucasian-American; CAV1 = caveolin-1; CTNNB1 = beta-catenin; MYH9 = myosin heavy chain-9; PRAD = prostate cancer adenocarcinoma; SRSF1 = serine/arginine-rich splicing factor 1; TCGA = The Cancer Genome Atlas; XRCC5 = X-ray repair cross-complementing protein 5

To determine the relevance of the 63 DEPs identified in the RC-77 cell line series in human prostate cancer specimens, we extracted protein and RNA expression data from TCGA PRAD cohort. We compared the protein and RNA expression of the 63 DEPs between African-American and Caucasian-American prostate cancer specimens; only caveolin-1, beta-catenin, myosin heavy chain-9, serine/arginine-rich splicing factor 1/splicing factor 2, double-stranded RNA-specific adenosine deaminase, and X-ray repair cross-complementing protein 5 had both protein and RNA data. X-ray repair cross-complementing protein 5 protein levels were significantly higher in African-American prostate cancer specimens than in Caucasian-American prostate cancer specimens (*p* < 0.05) (Fig. 4a). The RNA expression of caveolin-1 and myosin heavy chain-9 were significantly downregulated in African-American prostate cancer specimens compared to Caucasian-American prostate cancer specimens (*p* < 0.01 and *p* < 0.05, respectively) (Fig. 4b). After subtracting mRNA expression levels of non-malignant specimens from human prostate cancer specimens, caveolin-1 and beta-catenin mRNA expression levels were significantly higher in African-American prostate cancer patient specimens compared to Caucasian-American prostate cancer specimens (Fig. 5). As indicated by the negative RNA expression value, caveolin-1 was downregulated in African American prostate cancer specimens compared to African American non-malignant control specimens; on the contrary, beta-catenin was upregulated. Therefore, the reduction of caveolin-1 protein levels and the increased protein levels of beta-catenin seen in the tumorigenic RC-77 T/E cells were mirrored in the downregulation of caveolin-1 mRNA and upregulation of beta-catenin mRNA in African-American prostate cancer specimens.

## Discussion

The paired non-malignant and malignant African-American prostate epithelial cell lines RC-77 T/E and RC-77 N/E represent one of only a few cell lines derived from African-American prostate cancer patients [30]. E006AA, RC-165 N, and MDA-PCa 2a/2b are other African-American patient-derived cell lines. E006AA also has a highly tumorigenic derivative, E006AA-hT, and an associated stroma cell line, S006AA [27]. While the E006AA-hT model can be used to examine the differences between less and more highly tumorigenic cancers, it does not have a non-malignant paired epithelial cell line. The RC-165 N cell line is unique because it was derived from benign prostate tissue of an African-American male and was immortalized by telomerase [41]. This cell line is useful for understanding the functions of the androgen receptor in prostate epithelial cells. MDA-PCa 2a/2b cells are tumorigenic but differ in vivo and in vitro. These cell lines are a useful androgen sensitive model, but, unlike RC-77 cells, they do not have a paired non-malignant cell line from the same patient [29]. As RC-77 cell lines have epithelial-like characteristics, have functioning androgen receptors, and are immortalized with both a malignant and non-malignant pair, they represent a promising model for studying prostate cancer.

Here, we report the global proteomic characterization of RC-77 T/E and RC-77 N/E cell lines. Since RC-77 T/E cells are tumorigenic and RC-77 N/E cells are not, we analyzed DEPs between the two phenotypes. In overrepresentation analysis, GSEA, and SPIA, we consistently found that beta-catenin, alpha-actinin-1, integrin beta-1, integrin alpha-6, caveolin-1, laminin subunit gamma-2, CD44 antigen, and filamin-A expression levels contributed to the significance of the pathways highlighted in this report. Each of these proteins has structural roles or roles in cell adhesion, which explains why structural proteins were more prevalent among DEPs than could be expected by random chance and why many overrepresented pathways were related to cell adhesion (cell-cell or cell-matrix) or integrin signaling. Beta-catenin forms a complex with E-cadherin at adherens junctions to mediate cell-cell adhesion [42]. Alpha-actinin-1 forms focal adhesions, adherens junctions, tight junctions, and hemidesmosomes; forms cell-cell or cell-matrix contacts; and plays a scaffolding role for the cytoskeleton in a variety of signaling pathways [43]. Integrins interact with extracellular matrix (ECM) components to form cell-matrix attachments and propagate extracellular signals [44]. Caveolin-1 is an important component of caveolae, which are involved in molecular transport, cell adhesion, motility, and signal transduction [45, 46]. Laminin forms part of the basement membrane in some epithelial tissues and functions in adhesion, migration, invasion, and differentiation [47]. The glycoprotein CD44 antigen mediates cell adhesion and cytoskeleton binding through interactions with other proteins such as ankryin and ezrin, radixin, and moesin (ERM) proteins [48] and mediates hyaluronan-stimulated proliferation, apoptosis inhibition, cell motility, invasion [49]. Filamin-A cross-links actin filaments and serves as a scaffolding protein to organize the actin cytoskeleton [50], which affects cell motility, migration, and signaling [51].

Furthermore, expression levels of beta-catenin, caveolin-1, integrin beta-1, integrin alpha-6, CD44 antigen, and alpha-actinin-1 have been shown to differ by race. Here, we have shown higher beta-catenin protein levels in malignant RC-77 T/E cells compared to RC-77 N/E cells and that its mRNA is upregulated in African-American prostate cancer specimen compared to Caucasian-American specimen after subtracting the mRNA expression of race-specific non-malignant controls. These results are consistent with previous reports that beta-catenin is highly elevated in African-American prostate tumors compared to Caucasian tumors [16, 52]. Integrin alpha-6 and integrin beta-1 were downregulated in RC-77 T/E cells compared to RC-77 N/E cells, and integrins have been shown to be downregulated in African-American prostate cancer tissue compared to Caucasian specimens [12]. Thus, in these aspects, RC-77 T/E cells reflect in vivo characteristics of African-American prostate cancer and may be useful in the study of malignant transformation in African-American prostate tumors. While alpha-actinin-1 was upregulated in malignant RC-77 T/E cells, it was downregulated in African-American prostate cancer tissue compared to Caucasian specimens [12]. Our results also showed that caveolin-1 protein level was lower in malignant RC-77 T/E cells than non-malignant RC-77 N/E cells and that its mRNA expression was downregulated in African-American prostate cancer patient specimen compared to non-malignant African-American prostate specimen. After subtracting race-specific non-malignant RNA expression, caveolin-1 mRNA expression was higher in African-American prostate cancer patient specimens than in specimens from Caucasian-American patients. This result is in agreement with another study reporting elevated caveolin-1 protein expression in African-American prostate cancer specimens compared to Caucasian-American specimens [53]. African-American prostate cancer patients were also found to have higher rates of methylation of the CD44 gene [54], which was downregulated in malignant RC-77 T/E cells in this study.

To understand how differential expression of beta-catenin, caveolin-1, integrin beta-1, integrin alpha-6, CD44 antigen, and alpha-actinin-1 in RC-77 T/E cells may be related to phenotypic differences between RC-77 T/E and RC-77 N/E cell lines, we looked at the interactions between the DEPs using both STRING (to visualize direct interactions) and pathway analyses. First, the STRING network map revealed beta-catenin, integrin beta-1, and caveolin-1 in nodal positions, meaning these proteins may interact with several other DEPs in our dataset and may be a key regulator of the pathways highlighted in our results. For example, interaction between filamin-A and integrin beta-1 or caveolin-1 promotes migration, cell spreading, or metastasis, while interaction with other proteins results in inhibition of metastasis [51]. While filamin-A was upregulated in RC-77 T/E cells, integrin beta-1, caveolin-1 and vimentin, three of its binding partners that promote metastasis, were significantly downregulated. This is congruent with our knowledge that RC-77 T/E cells are derived from early stage primary prostate cancer (Gleason score 7) and are not metastatic [30]. Second, pathway analyses revealed that a common thread among the significant pathways was the inclusion of structural proteins, which could each be linked to invasion or migration of cells. “Tight Junction” and “Adherens Junction” pathways were enriched specifically in RC-77 T/E cells. Tight junctions are composed of claudin proteins, junctional adhesion molecules, integral membrane proteins, and cytoplasmic proteins, while adherens junctions are formed of cadherins and catenins [55]. Both hold together adjacent cells and help with structural and mechanical cell-cell integrity. The disruption of cell adhesion can facilitate the metastasis of tumor cells to secondary locations and lead to cell growth unchecked by contact inhibition [56]. The “Focal Adhesions” and “Proteoglycans in Cancer” pathways were significantly inhibited in RC-77 T/E cells. Proteoglycans in the tumor microenvironment associate with ECM proteins and affect proliferation, adhesion, and metastasis [57]. While the significance of the “Small Cell Lung Cancer” KEGG pathway may seem odd, it was highlighted in this dataset because of the role of ECM-receptor interactions and focal adhesions in cancer progression (see Additional file 10). “Cell Adhesion Molecules” and “ECM-Receptor Interaction” pathways, which were enriched in RC-77 N/E cells according to GSEA and shown to be significant by SPIA, were primarily flagged because of integrin expression.

## Conclusion

We detected 63 differentially expressed proteins between the malignant RC-77 T/E and the non-malignant RC-77 N/E cell lines, with 18 proteins uniquely detected in RC-77 T/E cells and 2 proteins uniquely detected in RC-77 N/E cells. The STRING network map revealed beta-catenin, integrin beta-1, and caveolin-1 in nodal positions, suggesting these proteins interact with several other DEPs and may be key regulators of the identified pathways. The “Tight Junction”, “Cell Adhesion Molecules”, “Adherens Junction”, “ECM-Receptor interaction”, “Focal Adhesion”, and “Proteoglycans in Cancer” pathways were shown to correlate with either RC-77 T/E or RC-77 N/E cells. Because structural proteins were overrepresented among DEPs and because several of the DEPs common to the significant pathways identified are structural proteins or have a structural role, our findings suggest that structural proteins may significantly contribute to the phenotypic differences between RC-77 T/E and RC-77 N/E cell lines. Based on data from both human prostate cell lines and limited patient specimens, our results indicate that differential expression of caveolin-1 and beta-catenin may be race- and prostate cancer-specific. A larger number of patients will be required to verify these findings. Although the RC-77 cell model may not be representative of all African-American prostate cancer due to tumor heterogeneity, it is a resource for studying prostate cancer initiation and progression.

## Additional files

Additional file 1: Operating Parameters for Mass Spectrometry Experiments. This text file provides the technical operating parameters for the mass spectrometry experiments

Additional file 2: MA Plot. This MA plot shows the data before (a) and after (b) transformation. The variances of the data remained similar before and after transformation, except for the larger average effects (> 40 in original scale). (TIFF 347 kb)

Additional file 3: KEGG Pathways Selected for Inclusion in All Pathway Analyses. These are the KEGG pathways used in Gene Set Enrichment Analysis and Signaling Pathway Impact Analysis. Pathways likely to have little relevance to prostate cancer (e.g., parasitic, bacterial, and viral infectious diseases; substance dependencies; and specific immune, neurodegenerative, and cardiovascular diseases) were excluded from the set

Additional file 4: TCGA PRAD DEPs Protein and mRNA Expression Data. This spreadsheet contains the patient demographics, tumor characteristics, and protein and mRNA expression data for the 24 age- and stage-matched African-American and Caucasian-American tumors used in this study. Additional sheets present the data of patients used for the non-malignant comparison and the expression values after subtracting the race-specific averaged values from the tumor expression values. (XLSX 23 kb)

Additional file 5: Proteins Identified in RC77T/E and RC-77 N/E Cell Lines. This table lists all protein assignments and raw spectral counts obtained by high-resolution electrospray tandem mass spectrometry (nLC-ESI-LIT-Orbitrap) for all biological replicates. Additional file 6: Processed Proteomics Data. This table contains the working dataset formed after processing the raw data. Processing included summing isoform data, rounding expression data up to the nearest whole number, and calculating log2 fold change ratios

Additional file 7: Reproducibility of Protein Fold Changes among Biological Replicates. This figure shows the log_2_ fold changes of corresponding biological replicates among RC-77 T/E and RC-77 N/E cell lines. The variations are well-controlled, as the majority of the proteins having fold changes less than 2 in both normal and tumor cell lines. (PDF 936 kb)

Additional file 8: Additional Analysis on Reproducibility of Protein Fold Changes between Paired Malignant and Non-Malignant Replicates. The differential expressions are stable across different pairs of tumor and non-malignant cell lines. (PNG 695 kb)

Additional file 9: Complete Gene Set Enrichment Analysis Results. This table lists the enriched gene sets identified from KEGG, BioCarta, and Reactome databases using Gene Set Enrichment Analysis. Positive enrichment scores correspond to enrichment in the malignant samples (RC-77 T/E). Negative enrichment scores correspond to enrichment in the non-malignant samples (RC-77 N/E). SIZE = total number of genes in pathway, ES = enrichment score, NES = normalized enrichment score, NOM p-val = unadjusted probability of enrichment, FDR q-val = false discovery rate-adjusted probability. Additional file 10: Complete Signaling Pathway Impact Analysis Results. This table presents the complete results of Signaling Pathway Impact Analysis. For each pathway, a link to a pathway diagram highlighting differentially expressed proteins in red is provided. ID = KEGG ID, pSize = pathway size, NDE = number of differentially expressed proteins in pathway, pNDE = probability of overrepresentation, tA = total accumulated perturbation, pPERT = probability of perturbation, pG = combined global probability of overrepresentation and perturbation, pGFdr = false-discovery rate-adjusted global probability, pGFWER = familywise error rate-adjusted global probability

## Abbreviations

DEP: Differentially expressed protein
ECM: Extracellular matrix
FDR: False discovery rate
GO: Gene Ontology
GSEA: Gene Set Enrichment Analysis
KEGG: Kyoto Encyclopedia of Genes and Genomes
PANTHER: Protein ANalysis THrough Evolutionary Relationships
pGFdr: False discovery rate-adjusted global probability
PRAD: Prostate adenocarcinoma
SPIA: Signaling Pathway Impact Analysis
STRING: Search Tool for the Retrieval of Interacting Genes/Proteins
TCGA: The Cancer Genome Atlas
