# Supplementary material for: Proteomic characterization of paired non-malignant and malignant African-American prostate epithelial cell lines distinguishes them by structural proteins
Source: BMC Cancer. 2017 Jul 11;17:480. doi: 10.1186/s12885-017-3462-7 (PMC5504803; doi:10.1186/s12885-017-3462-7)
Supplement: Supplementary file 1 — MA Plot. This MA plot shows the data before (a) and after (b) transformation. The variances of the data remained similar before and after transformation, except for the larger average effects (> 40 in original scale). (TIFF 347 kb) [file 12885_2017_3462_MOESM1_ESM.tif]

Operating Parameters for Mass Spectrometry Experiments
For protein expression analysis by high-resolution electrospray tandem mass spectrometry, an externally calibrated Thermo LTQ Orbitrap Velos (nLC-ESI-LIT-Orbitrap) mass spectrometer was used with the following parameters. A 2-cm, 100-ìm internal diameter trap column (SC001 Easy Column from Thermo-scientific) was followed by a 10-cm analytical column of 75-ìm internal diameter (SC200 Easy Column from Thermo-scientific). Both trap column and analytical column had C18-AQ packaging. Separation was carried out using Easy nanoLC II (Thermo-Scientific) with a continuous, vented column configuration. A 2-ìL (200 ng) sample was aspirated into a 20 ìL loop and loaded onto the trap. The flow rate was set to 300 nL/min for separation on the analytical column. Mobile phase A was composed of 99.9 H2O (EMD Omni Solvent), and 0.1% formic acid  and mobile phase B was composed of 99.9% ACN,  and 0.1% formic acid. A 1 h linear gradient from 0% to 45% B was performed. The liquid chromatography eluent was directly nanosprayed into an LTQ Orbitrap Velos mass spectrometer (Thermo Scientific). During the chromatographic separation, the LTQ Orbitrap Velos was operated in a data-dependent mode and under direct control of the Xcalibur software (Thermo Scientific). The MS data were acquired using 10 data-dependent collisional-induced-dissociation MS/MS scans per full scan. Samples were run in technical triplicate to enable normalization and statistical comparisons between samples.
