# Supplementary material for: Proteomic characterization of paired non-malignant and malignant African-American prostate epithelial cell lines distinguishes them by structural proteins
Source: BMC Cancer. 2017 Jul 11;17:480. doi: 10.1186/s12885-017-3462-7 (PMC5504803; doi:10.1186/s12885-017-3462-7)
Supplement: Supplementary file 3 — Reproducibility of Protein Fold Changes among Biological Replicates. This figure shows the log2 fold changes of corresponding biological replicates among RC-77 T/E and RC-77 N/E cell lines. The variations are well-controlled, as the majority of the proteins having fold changes less than 2 in both normal and tumor cell lines. (PDF 936 kb) [file 12885_2017_3462_MOESM3_ESM.pdf]

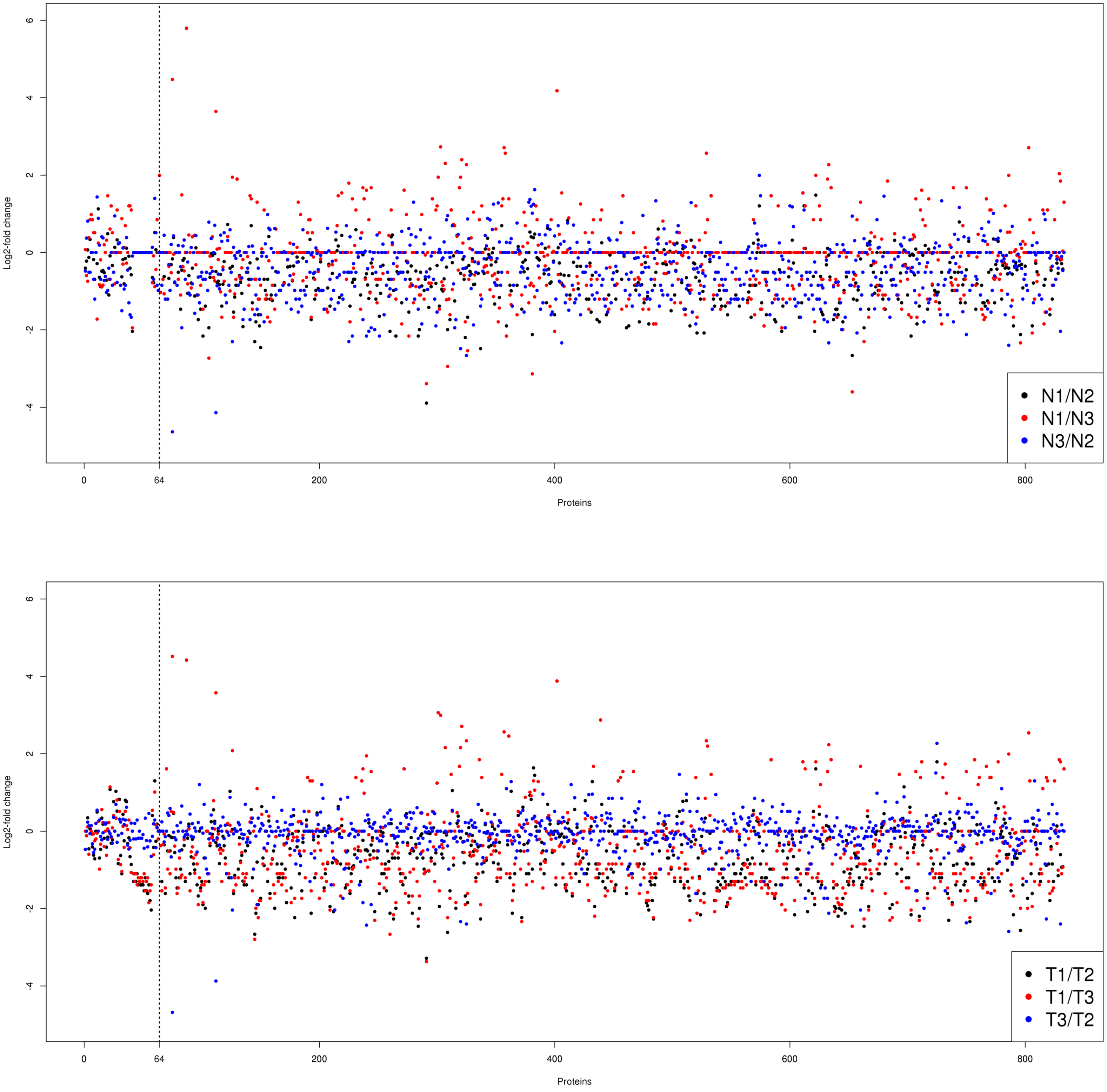

Reproducibility of Protein Fold Changes Among Biological Replicates. The log_2 fold changes were well controlled, and the majority of the proteins across biological replicates had log_2 fold change less than 2. Dots represent the log_2 fold change for each protein. N = normal, T= tumor, FC = fold change.
