# Supplementary figures and images for: Proteomic characterization of paired non-malignant and malignant African-American prostate epithelial cell lines distinguishes them by structural proteins
Source: BMC Cancer. 2017 Jul 11;17:480. doi: 10.1186/s12885-017-3462-7 (PMC5504803; doi:10.1186/s12885-017-3462-7)

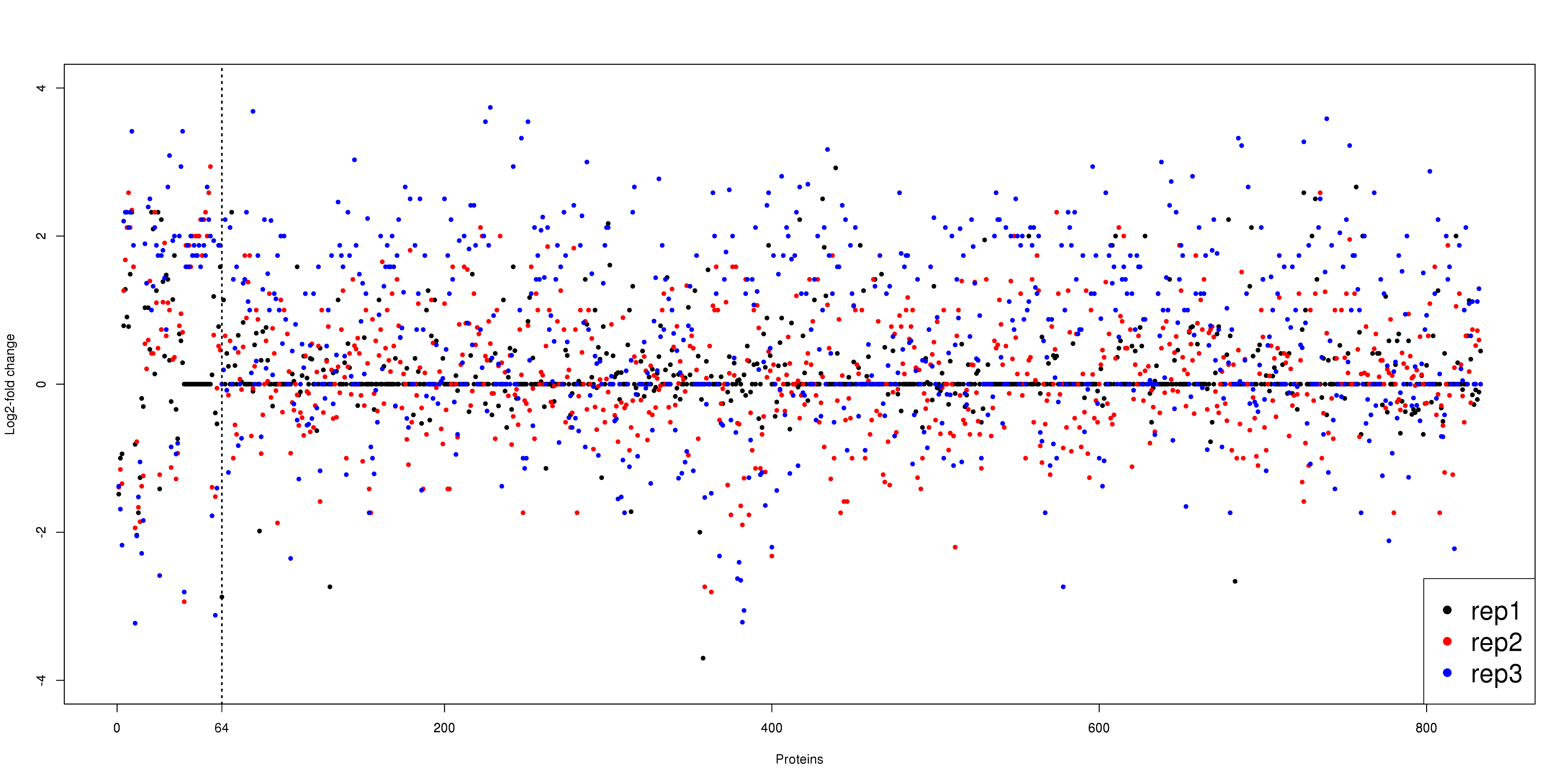

Supplement: Supplementary file 4 — Additional Analysis on Reproducibility of Protein Fold Changes between Paired Malignant and Non-Malignant Replicates. The differential expressions are stable across different pairs of tumor and non-malignant cell lines. (PNG 695 kb) [file 12885_2017_3462_MOESM4_ESM.png]
